# Observational Bias during Nutrition Surveillance: Results of a Mixed Longitudinal and Cross-Sectional Data Collection System in Northern Nigeria

**DOI:** 10.1371/journal.pone.0062767

**Published:** 2013-05-03

**Authors:** Emmanuel Grellety, Francisco J. Luquero, Christopher Mambula, Hassana H. Adamu, Greg Elder, Klaudia Porten

**Affiliations:** 1 Epicentre, Paris, France; 2 Médecins Sans Frontières, Paris, France; 3 Ministry of Health, Jigawa state, Nigeria; Kenya Medical Research Institute - Wellcome Trust Research Programme, Kenya

## Abstract

**Background:**

The Sahel is subject to seasonal hungry periods with increasing rates of malnutrition. In Northern Nigeria, there is no surveillance system and surveys are rare. The objectives were to analyse possible observational bias in a sentinel surveillance system using repeated mixed longitudinal/cross-sectional data and estimate the extent of seasonal variation.

**Methods:**

Thirty clusters were randomly selected using probability proportional to size (PPS) sampling from Kazaure Local Government Area, Jigawa State. In each cluster, all the children aged 6–59 months within 20 randomly selected households had their mid-upper arm circumference measured and were tested for oedema. The surveys were repeated every 2 or 4 weeks. At each survey round, three of the clusters were randomly selected to be replaced by three new clusters chosen at random by PPS. The seasonal variation of acute malnutrition was assessed using cyclical regression. The effect of repeated visits to the same cluster was examined using general linear mixed effects models adjusted for the seasonal change.

**Results:**

There was a significant seasonal fluctuation of Global Acute Malnutrition (GAM) with a peak in October. With each repeat survey of a cluster, the prevalence of GAM decreased by 1.6% (95% CI: 0.4 to 2.7; p = 0.012) relative to the prevalence observed during the previous visit after adjusting for seasonal change.

**Conclusions:**

Northern Nigeria has a seasonal variation in the prevalence of acute malnutrition. Repeated surveys in the same cluster-village, even if different children are selected, lead to a progressive improvement of the nutritional status of that village. Sentinel site surveillance of nutritional status is prone to observational bias, with the sentinel site progressively deviating from that of the community it is presumed to represent.

## Introduction

The population of Nigeria is over 170 million (2012) [1] and malnutrition is estimated to be the immediate or underlying cause of more than 50% of deaths among children under 5 years [2].

Seasonal peaks and fluctuating levels of acute malnutrition with annual threats of famine are characteristic of several Sahelian countries of West and Central Africa [3]. However this pattern has not been adequately examined in Northern Nigeria which is considered to be in the southern Sahel. The “hunger season” which affects the rest of the Sahel annually would also be expected to affect Northern Nigeria with increased levels of acute malnutrition and associated increases in mortality.

Nutrition information systems use several different sources of data to provide information [4]. These are usually grouped into four categories for young children: 1) repeated surveys; 2) sentinel sites; 3) data collected in health facilities (e.g. growth monitoring); and 4) data on admissions to feeding programs [5]. Many nutrition surveillance systems also collect food security, agricultural, economic, climatic and other contextual information.

In terms of nutrition surveillance per se, current methods are affected by different types of bias. Hospital and health facility based reporting systems are rarely representative of the population [6]. They are usually biased because of limited access and cost of health services, poor quality of measurements and varying case definitions. Repeated surveys require a higher level of technical expertise, can be very costly [7] and are usually performed infrequently. The bias arises where there are seasonal changes because it gives a “snapshot” of the situation at a particular point in time when the prevalence of nutritional disorders can change markedly with season. Thus, if a survey takes a long time to complete those areas measured during the “hungry season” will appear to be more affected than those measured before or after that period giving a false impression of the status of different areas. Such infrequent surveys are not appropriate in a nutritional emergency or impending famine when data are required immediately and trends in nutritional status need to be observed [8].

Sentinel site monitoring also has the potential to be biased because the sites chosen may progressively differ from the rest of the community due to the inputs of the survey teams; this can be by giving education, advice and counselling, treating illness where it is observed by the teams and referral of any malnourished child to a treatment program or by providing employment and spending funds within the community [9].

While no single method is ideal, frequently collected data that are representative of the whole community are most easily understood and interpreted and explain why sentinel site surveillance has been most frequently adopted [8]. However, the degree to which sentinel surveillance is subject to bias and how any such bias evolves with time has not been adequately examined.

In April 2009, the Commissioners and Secretary of State of the Federal Republic of Nigeria declared that malnutrition was an emergency. For this reason in April 2010, Médecins Sans Frontières (MSF) in collaboration with Epicentre and the Ministry of Health started a therapeutic feeding programme for severe acute malnutrition accompanied by prevalence studies through repeated surveys in Kazaure Local Government Area (LGA) within Jigawa State. The objective was to allow timely detection of severe acute malnutrition and respond to any nutritional emergency that arose. The system had a unique design using repeated mixed longitudinal/cross sectional data collection to achieve the advantages of sentinel site surveillance whilst minimising its possible disadvantages. We report the results found with this novel design and estimate the bias that arises from repeated observations of the same cluster.

## Methods

### Ethics

This study was based on analysis of routinely collected, patient monitoring data from the programmes for acute malnutrition. A Memorandum of Understanding to implement and analyse the surveillance system was signed with the Ministry of Health. In agreement with the Ministry of Health clinical and therapeutic patient data are routinely collected for patient and programme monitoring; as such, no formal ethics approval from institutional review boards and/or written patient consent were required by either the Ministry of Health, Nigeria, or the rules of the MSF Ethical Review Board. Local health authorities were informed of the potential publication of findings, with approval from the Nigerian health authorities. We followed the Declaration of Helsinki, aiming to provide assurance that the rights, integrity, and confidentiality of participants were protected [10]. We obtained oral consent from participants or their parents or guardians. We ensured privacy and confidentiality in the data collected from the participants both during and after the conduct of the study. We entered and analyzed all information anonymously and findings were shared with our partners in the health ministries.

### Study Design

A population-based multi-stage cluster sampling surveillance system was used. The system was a combination of longitudinal and cross sectional data collection, with progressive random replacement of sites to avoid and assess any “drift” of the surveyed villages from being representative of the population at large. The repeated cluster survey approach based on measuring mid-upper-arm-circumference (MUAC) and examining for bilateral oedema was chosen due to the ease and speed of obtaining these data, thus reducing the cost and increasing the feasibility of frequently repeated surveys. Thirty clusters were randomly selected using probability proportional to size (PPS) sampling [11]. The surveys were repeated on a fortnightly basis (from June 2010 to April 2011) and thereafter on a monthly basis (from May 2011 to February 2012) to avoid community and team fatigue.

At each survey round, three of the 30 clusters (10%) from the previous round were chosen at random to be replaced. The 3 clusters to be replaced were returned to the sampling frame so that the current sampling frame included all villages not used as clusters in the previous round plus the three excluded clusters, but excluding the 27 clusters already selected to be surveyed. This sampling frame was then used to select the 3 replacement clusters using PPS.

The sampling frame was based on the 2010 projection of 2006 census data.

As this design combines elements of sentinel site surveillance and repeated PPS surveys we refer to this design as “hybrid nutritional surveillance”.

At each round a minimum of 20 households were selected randomly from each cluster using the EPI 2 method [12]: thus, a household that was previously sampled may or may not be sampled on a subsequent occasion. The sample size was calculated based upon 5 person households with one eligible child, a maximum expected prevalence of 15%, a design effect of 2.0, a non-response rate of 10% and a precision of 5% to be 527 households and was rounded up to 600 (30×20).

A household was defined as a group of people sleeping and eating together from a common cooking pot. Within each household all the children aged between 6 to 59 months had their, age, sex and whether they were receiving treatment for severe acute malnutrition (SAM) recorded; their MUAC was measured and they were tested for oedema. At cluster level, GPS position, ethnic group and the price of the main food items were also collected. An independent PPS survey was conducted quarterly, using the same sampling frame where weight and height were also measured and a 3 month retrospective mortality estimate obtained (data not shown).

### The Survey Teams

Physically fit home visitors, who could speak English and the local language, read, write and count accurately, were recruited from both the Ministry of Health and MSF. There were 3 teams of two home visitors (one male and one female), each surveying two clusters per day. The teams were not assigned to particular clusters to avoid systematic bias. They were closely followed by the supervisor during all data collection. They attended five days training focusing on the aims of the surveillance system, the importance of data collection and how to avoid selection, information and measurement biases; followed by practical training taking anthropometric measurements and testing for oedema with a formal standardisation test. Refresher training was repeated several times during the surveillance follow-up and the standardisation test to assess the precision and accuracy of the measurements [13] was repeated quarterly and whenever a new team member was recruited.

### Data Collection

The ages of children were ascertained with the help of a local events calendar. If a child’s age was uncertain, his/her eligibility was judged using a stick marked at 60, 65, 75, 85, 95 and 110 cm – children 65 to 110 cm were then included in the sample. Children meeting the criteria for severe acute malnutrition (SAM: MUAC<115 mm and/or bilateral oedema) were referred for treatment.

Single data entry was performed on ENA Delta [14]; a plausibility check was run to identify duplicate entries, missing values, age distribution, sex and age ratio, digit preference, design effect and if cases were randomly distributed or aggregated over the clusters by calculation of the index of dispersion and comparison with the Poisson distribution for global acute malnutrition (GAM) and SAM. MUAC outliers were identified using the SMART [14] procedure of flagging those values which were more than 3 standard deviations from the mean. This check was performed for the overall survey and by team on a daily bases in order to exclude/replace enumerators without the requisite skill.

Some of the clusters, which had been previously removed, were subsequently re-selected. As the previous intervention in the cluster could influence the data collected subsequently, only data collected during the first selection of a cluster are included in this analysis.

### Variables

Three variables were considered as outcomes: (i) absolute value of the child’s MUAC; (ii) GAM prevalence defined as MUAC<125 mm or bilateral oedema; and (iii) SAM prevalence defined as MUAC<115 mm or bilateral oedema. MUAC was measured as a continuous variable and GAM and SAM prevalence as dichotomous variables.

Two main predictor variables of interest were (1) a time variable modelled cyclically to assess seasonal variation and (2) a variable specifying the number of times that each particular cluster had been surveyed (to assess effect of repeated visits to the same cluster). Additional independent variables were age, sex and ethnic group of the children.

### Analysis

We calculated the difference in the MUAC and the prevalence ratios (PR) of GAM and SAM in order to analyse the relation of these three outcomes with the different predictor variables. The PR is used to examine the relative change in prevalence. For dichotomous variables (e.g. sex), the PR represents the ratio of prevalence between the two groups. For continuous variables (e.g. the number of surveillance visits per cluster), the PR represents the relative increase/decrease of the prevalence for each unit in the continuous variable.

First, univariate analyses were conducted; the crude effect of the two predictor variables was assessed for each outcome. Second, multivariate analyses adjusted by age, sex and ethnic group were performed.

We used general linear mixed effects models (GLMM) to take into account the repeated measures and multi-stage design of the study. We considered three levels in the model: the individual, the clusters and the number of surveillance visits per cluster. The GLMM equation was as follows:
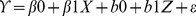
where Y is the outcome (dependent) variable, and X are the main predictor (independent) variables (the number of surveillance visits per cluster and the seasonality), Z are the variables with random effects, the fixed effects: β0 is the population intercept, β1 is the population slope, and random effects: b0 is the study intercept and b1 is the study slope (the cluster and the number of surveillance visits per cluster).

We analysed the seasonal variations using a cyclical regression that included one year cycle (365 days). The seasonal pattern was modelled as follows (fixed effects part of the model):
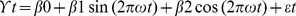
where Yt is the outcome variable (MUAC mean, GAM or SAM), t is time in days, ω is frequency (ω = 1/365), β0 is intercept and β1, β2, are regression parameters, and εt is the error term. We used one cycle to account for the single peak seen during the nutrition surveillance. Using the estimate for the regression parameters, we calculated peak timing and intensity based on the δ-method [14]–[15]. The relative intensity of the peak was calculated by dividing the expected seasonal maximum value by the expected seasonal minimum value.

In the multivariate model the two main predictors were included and additional terms were included for age, sex and ethnic group. A Gaussian distribution was assumed to model the MUAC and a binomial distribution for GAM and SAM with a log link and a robust estimation of parameters. Main interactions between the predictors were tested and linear, quadratic and cubic relationships were assessed between the number of surveillance visits per cluster and the different outcomes.

The fit of the GLMM was assessed using the variance of the Pearson residual and regression parameters were tested at α = 0.05 significance level.

Point coverage was calculated as the number of SAM children identified that were enrolled in the feeding program expressed as a percentage of the total number of SAM children identified. Period coverage was calculated as the number of children surveyed who were enrolled in the feeding program (whether or not they were still SAM) expressed as a percentage of the number of cases of SAM identified plus the number enrolled in a feeding program who were no longer SAM.

All analyses were performed with Stata 10 software (Stata Corporation, College Station, TX), using the generalized linear latent and mixed model (GLLAMM) framework [16] and the package “spatstat” of R software v.2.9.2 [17] for the spatial representation using a Gaussian kernel function.

## Results

### Description of Children Included in the Nutritional Surveillance System

The surveillance system was conducted between 21^st^ June 2010 and 17^th^ February 2012; there were 16,466 measurements of children included in the analysis; 2,425 measurements of children from re-selected clusters were not included; the reselected cluster’s children were not significantly different for the variables in table 1 from those included in the analysis. The mean age of these children was 34.0 months (SD 16.7 months); there were slightly fewer girls (48.8%) than boys; 71.4% were ethnically Hausa and the remainder Fulani (28.6%). The overall mean prevalence of acute malnutrition over the whole period was 8.4% GAM and 1.8% SAM with a point coverage in the therapeutic feeding program of 30.3% (Table 1).

**Table 1:** Characteristics of children included in the nutritional surveillance system from clusters that were not re-selected between June 2010 and February 2012 in Kazaure LGA, Jigawa State, Nigeria.

Child characteristics	N	%
Age, months (N = 16453)		
6 to 11	1698	10.32
12 to 23	3333	20.26
24 to 35	3115	18.93
36 to 47	3250	19.75
48 to 59	5057	30.74
Gender (N = 16466)		
Boys	8425	51.17
Girl	8041	48.83
Height categories (N = 16452)		
60–<85 cm	8616	52.37
85–<110 cm	7836	47.63
Ethnic group (N = 16466)		
Hausa	11750	71.36
Fulani	4716	28.64
Acute malnutrition (N = 16466)		
MUAC<125 mm or bilateral oedema	1388	8.42
Boys	626	7.43
Girls	762	9.47
MUAC<115 mm or bilateral oedema	300	1.82
Boys	133	1.57
Girls	167	2.07
Bilateral oedema	21	0.12
Point coverage	91	30.33
Period coverage	686	76.65

### Description of the Nutritional Surveillance System

**Figure 1:**
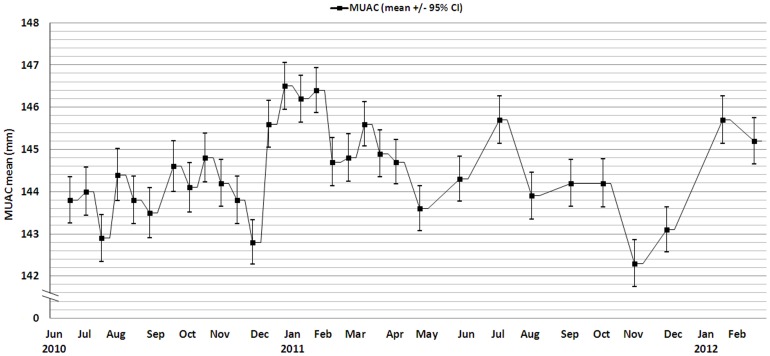
MUAC mean for children aged from 6 to 59 months between June 2010 and February 2012, Kazaure LGA, Jigawa State, Nigeria.

**Figure 2:**
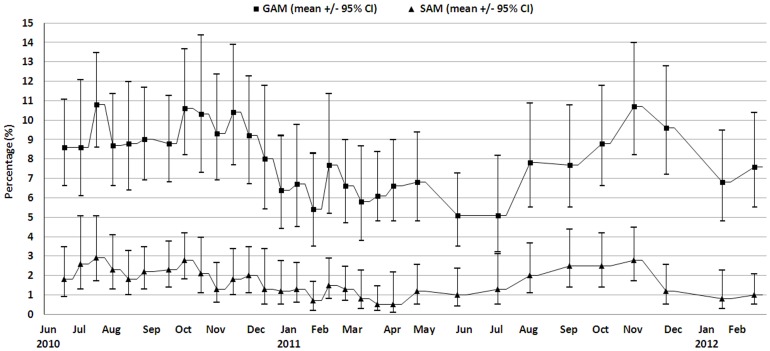
GAM and SAM prevalence based on MUAC and/or bilateral oedema for children aged from 6 to 59 months between June 2010 and February 2012, Kazaure LGA, Jigawa State, Nigeria.

A total of 31 cross sectional surveys were performed; 21 fortnightly from June 2010 to April 2011 and then 10 monthly until February 2012. A total of 108 different clusters were surveyed; 19 were re-selected more than once and the data from second and subsequent surveys of the same cluster excluded from the analysis. Figure 1 shows the mean absolute MUAC and Figure 2 the prevalence of GAM and SAM during this period.

**Figure 3:**
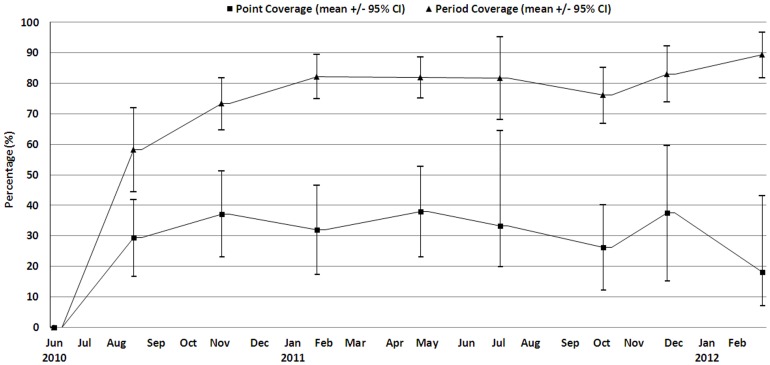
Program point and period coverage based on MUAC <115 mm or bilateral oedema for children aged from 6 to 59 months between June 2010 and February 2012, Kazaure LGA, Jigawa State, Nigeria.

The point and period program coverage was computed every four weeks and are presented in Figure 3.

### Univariate and Multivariate Analysis

**Figure 4:**
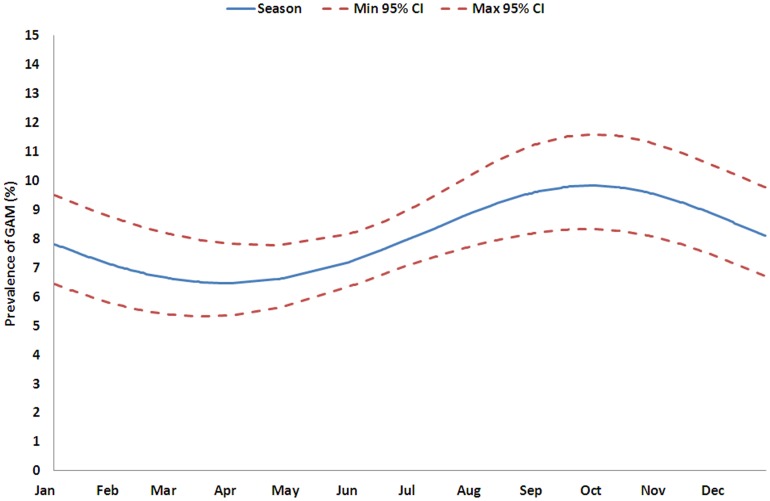
Prediction of seasonal variation of the GAM using a Binomial GLMM, Kazaure LGA, Jigawa State, Nigeria.

**Figure 5:**
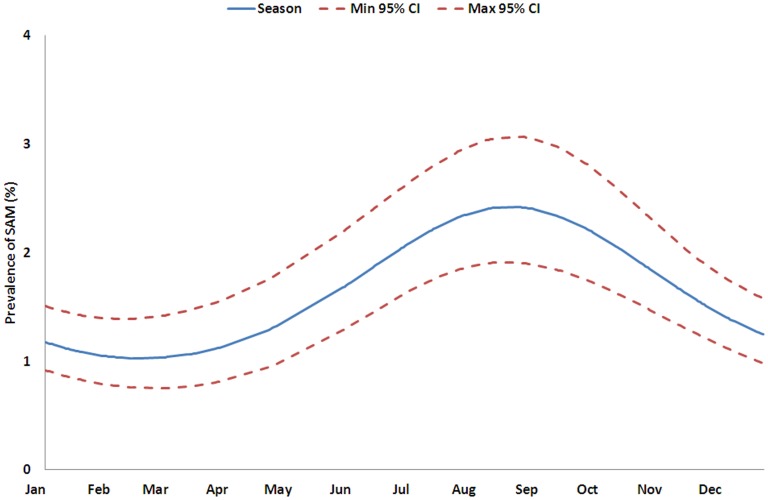
Prediction of seasonal variation of the SAM using a Binomial GLMM, Kazaure LGA, Jigawa State, Nigeria.

Table 2 shows the results of the univariate and multivariate linear analysis of the absolute MUAC. In the univariate analysis MUAC varied significantly with the number of times the cluster was surveyed, and also with age, sex, ethnic group and seasonal pattern. These factors remained significant in the multivariate analysis showing that they each made an independent contribution to the variance. The number of times a cluster had been visited was significantly associated with an increase in the average MUAC between 0.006 mm and 0.124 mm per visit (95% CI). Table 3 shows the variations in GAM and SAM expressed as the relative change in prevalence. The GAM varies significantly with the number of times the cluster has been surveyed, age, sex, ethnic group and season both with the univariate and multivariate analysis. The prevalence of SAM varied with the number of times the cluster has been surveyed, age, sex, ethnic group and season in the univariate analysis, but only with sex, age, and seasonality in the multivariate analysis. Thus, as a particular cluster village is repeatedly visited, the prevalence of acute malnutrition decreases linearly. The PR per surveillance visit was 0.98 (95% CI: 0.973 to 0.996; p = 0.012) for GAM and 0.99 (95% CI: 0.962 to 1.017; p = 0.475) for SAM compared with the previous visit (Table 4). This means that the observed prevalence decreases by 1.6% (95% CI: 0.4 to 2.7, GAM) and 1.1% (95% CI: 0.0 to 3.8, SAM) relative to the prevalence observed during the previous visit after adjusting for seasonal change. The seasonal changes in GAM and SAM prevalence were significant; figures 4 and 5 show the predicted variation over one year. GAM prevalence reaches a maximum between July and December with a peak in October and SAM between June and November with a peak in September (Table 3 and 4). The relative intensity of the peaks is 1.5 and 2.4 respectively.

**Table 2:** Regression parameters, confidence intervals and p-value using a Gaussian GLMM, Kazaure LGA, Jigawa State, Nigeria.

	Univariate models			Multivariate model		
	Estimate	(95% CI)	*P*	Estimate	(95% CI)	*P*
Absolute MUAC*		(0.006; 0.124)	0.030			
Surveillance visits per cluster	0.065			0.060	(0.005; 0.114)	0.031
Sex (boys vs. girls)	0.743	(0.328; 1.158)	<0.001	0.989	(0.653; 1.325)	<0.001
Age (months)	0.455	(0.445; 0.465)	<0.001	0.439	(0.429; 0.449)	<0.001
Ethnic group (Hausa vs. Fulani)	1.784	(1.118; 2.451)	<0.001	2.221	(1.624; 2.817)	<0.001
Seasonal pattern (cos)	−0.312	(−0.744; 0.118)	0.155	−0.228	(−0.607; 0.151)	0.238
Seasonal pattern (sin)	−0.568	(−1.010; −0.127)	0.012	−0.619	(−1.005; −0.234)	0.002

Univariate and multivariate analysis (N = 16,453).

* Models take the absolute MUAC as the dependent variable with cluster and number of surveillance visit per cluster introduced as random-intercepts.

**Table 3:** Prevalence ratio, confidence intervals and p-value using a Binomial GLMM, Kazaure LGA, Jigawa State, Nigeria.

	Univariate models			Multivariate model		
	PR	(95% CI)	*P*	PR	(95% CI)	*P*
GAM*		(0.967; 0.991)	0.001			
Surveillance visits per cluster	0.979			0.984	(0.973; 0.996)	0.012
Sex (boys vs. girls)	0.778	(0.703; 0.861)	<0.001	0.745	(0.680; 0.816)	<0.001
Age (months)	0.929	(0.925; 0.933)	<0.001	0.928	(0.924; 0.932)	<0.001
Ethnic group (Hausa vs. Fulani)	0.821	(0.722; 0.934)	0.003	0.741	(0.676; 0.812)	<0.001
Seasonal pattern (cos)	0.946	(0.868; 1.032)	0.216	0.928	(0.857; 1.004)	0.066
Seasonal pattern (sin)	1.223	(1.118; 1.339)	<0.001	1.224	(1.128; 1.328)	<0.001
SAM**						
Surveillance visits per cluster	0.969	(0.944; 0.996)	0.025	0.989	(0.962; 1.017)	0.475
Sex (boys vs. girls)	0.734	(0.582; 0.925)	0.009	0.701	(0.557; 0.882)	0.002
Age (months)	0.924	(0.914; 0.993)	<0.001	0.923	(0.914; 0.933)	<0.001
Ethnic group (Hausa vs. Fulani)	0.951	(0.722; 1.253)	0.725	0.848	(0.646; 1.114)	0.238
Seasonal pattern (cos)	1.177	(0.985; 1.406)	0.072	1.157	(0.968; 1.384)	0.108
Seasonal pattern (sin)	1.487	(1.222; 1.808)	<0.001	1.491	(1.231; 1.807)	<0.001

Univariate and multivariate analysis (N = 16,453).

* Models take GAM as the dependent variable with cluster and number of surveillance visit per cluster introduced as random-intercepts.

** Models take SAM as the dependent variable with cluster and number of surveillance visit per cluster introduced as random-intercepts.

**Table 4:** Estimated peak timing from periodic regression for the nutritional surveillance between June 2010 and February 2012, Kazaure LGA, Jigawa Satate, Nigeria.

	Expected maximum (peak)			Expected minimum (nadir)			Relative Intensity
	Week (/52)	Seasonal value (%)	(95% CI)	Week (/52)	Seasonal value (%)	(95% CI)	
GAM	40	9.8	(8.3; 11.6)	14	6.5	(5.3; 7.8)	1.51
SAM	35	2.4	(1.9; 3.1)	8	1.0	(0.8; 1.4)	2.35

We did not find significant interactions between the predictors for the three outcomes. In addition, we assessed quadratic and cubic relationships between the number of surveillance visits per cluster and the different outcomes, but these were not significant.

**Figure 6:**
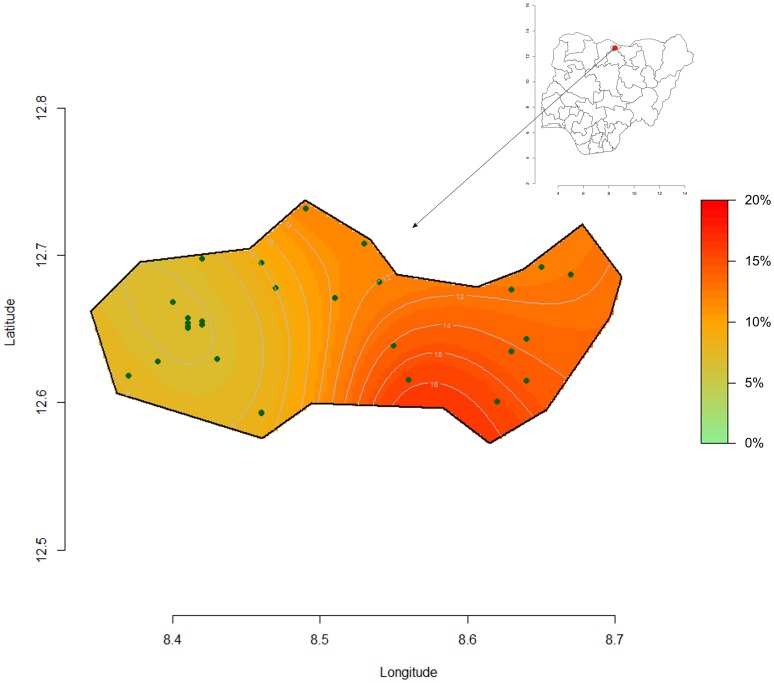
Spatial clusters representation of GAM week 40 at the peak period, Kazaure LGA, Jigawa State, Nigeria.

**Figure 7:**
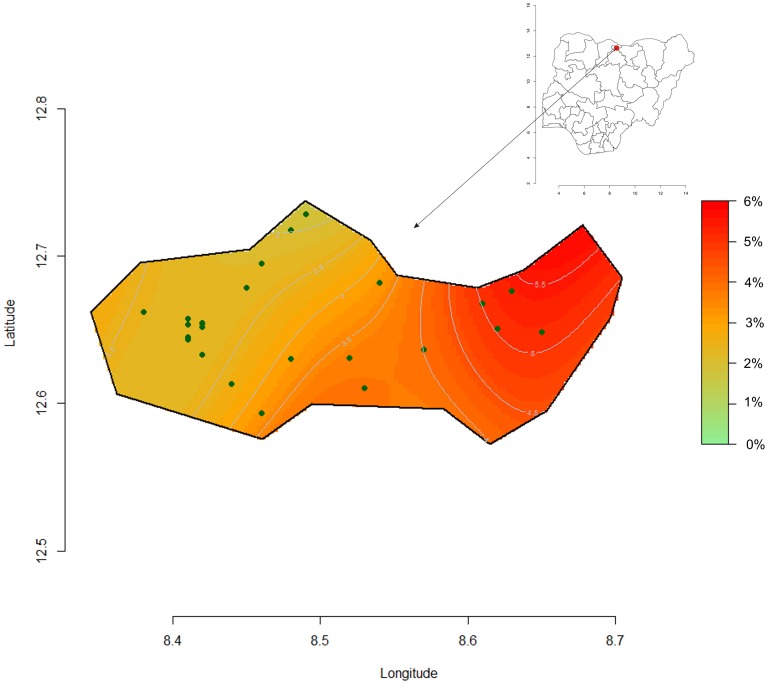
Spatial clusters representation of SAM week 35 at the peak period, Kazaure LGA, Jigawa State, Nigeria.

Figures 6 and 7, shows the computed spatial prevalence contours of GAM and SAM at peak prevalence (week 40 and 35). The prevalence appears to be highest in eastern Kazaure LGA with a steep gradient from very low to “emergency” levels.

## Discussion

This study shows that sentinel site surveillance is prone to observational bias when used to monitor changes in nutritional status of a community. A cross-sectional “hybrid” design would mitigate such bias whilst retaining the advantages of obtaining longitudinal data from a sentinel site. The study also showed a seasonal variation in the prevalence of acute malnutrition in Northern Nigeria throughout the year.

The Famine Early Warning Systems Network (FEWS Net) predicts the lean season in Northern Nigeria to be from July to September [18] using the seasonal calendar. The present data shows the highest prevalence of GAM between July and December, and for SAM between June and November. Both GAM and SAM are expected to peak concurrently. The model predicts the peak of SAM slightly before that of GAM; however, this should be interpreted with caution considering that only two complete seasons are included in the analysis, the difference is small and SAM children were offered treatment as soon as they were identified. The data corroborate FEWS-Net predictions and confirm that GAM and SAM prevalence rates change concurrently with the seasonal calendar and are not “trailing indicators”. Theoretically, if the mean MUAC decreases and the distribution remains the same, the proportionate increase in SAM will be greater than GAM. This was observed despite the fact that the SAM children were offered treatment and the moderately malnourished children were not treated. This could be explained by the relatively low point coverage of the treatment program and the extensive sharing of the therapeutic food documented elsewhere [19].

The data show that there is a significant improvement of the nutritional status of the children within survey villages, relative to the whole population surveyed, as they are repeatedly surveyed. The effect of repeated surveillance visits per cluster was significant for both absolute MUAC and GAM in both the univariate and multivariate analyses. With SAM as the outcome variable it ceased to be significant with multivariate analysis. This could either be due to relatively small number of children that developed SAM with loss of statistical power or because the SAM children were offered treatment.

Although the point coverage was 30%, the period coverage shows that up to 80% of the previously SAM children were at various stages of recovery and most were no longer classified as SAM. Even though there were few SAM children, their treatment could have changed the mean MUAC found as the clusters were repeatedly surveyed. Furthermore, sharing of the therapeutic food [19] given only in the surveyed clusters could have increased the MUAC of children who had not presented with SAM. We suggest that if the larger number of moderately malnourished children (MAM) had also been offered treatment the effect upon the mean MUAC of the “sentinel clusters” would have led to a much greater bias. At each surveillance visit there is a relative decrease of 1.6% (95% CI: 0.4 to 2.7) GAM prevalence. Thus, for example, if the real population prevalence in the area is 10% GAM, the “sentinel site effect” after 10 visits to the same cluster would result in a bias so that this sentinel cluster would show 8.6% instead of 10% GAM prevalence. Sentinel site surveillance systems normally sample the sentinel villages repeatedly over prolonged periods, sometimes many years. This finding confirms that sentinel site monitoring, as a surveillance system for nutritional status, can lead to considerable underestimation of the true situation within the population.

However, sentinel site surveillance has many advantages. The households in chosen villages can be mapped so that a random sample can be chosen rapidly and accurately; community members become familiar with the system and the data collectors. For these reasons, surveys at sentinel sites are relatively rapid and less costly than those which select new sites at each round. However, malnourished children within the village must be referred for treatment for ethical reasons, and this in time should modify the nutritional status of the children within that village, particularly if there is sharing of the therapeutic food [19]. Furthermore, the team itself will offer advice and referral of sick children, feed-back data to the village elders which will sensitise them to the nutritional problems within the village and if the team offers incentives to informants or spends money to purchase food or other items this has the potential to change the economics of the village. We were not able to assess the relative effects of these possible factors to explain our findings. Apprehension bias whereby direct interaction of the team with the children and their families could affect a measurement itself is unlikely to be a significant factor in measurement of MUAC, unlike other measures such as blood pressure or respiration rate [20]. There was no systematic change with time when repeated measures of MUAC were taken from the same children in the standardization tests. It is clear that, with time, sentinel sites may cease to be representative of the community and gradually result in erroneous conclusions with respect to community nutritional status. The magnitude of this bias has not to our knowledge been previously examined in a community subject to annual nutritional stress. If a sentinel site design is to be used it is recommended that sites should be replaced wherever this is feasible. It should be emphasised that although the same villages were surveyed, the children within that village were selected at random at each visit, so that different children were usually measured in each cluster at each visit. It is anticipated that if the same children had been measured from “sentinel households” the effect of repeated visits would have been far greater. It is a tenet of physical science that observing an object changes that object (Heisenberg's uncertainty principle); the present study shows that this applies particularly to repeated measurements of nutritional status and emphasises a potential problem with extrapolation from longitudinal data taken from the same individual, household, center or village to a community.

To our knowledge, this is the first time that a study has quantified the extent of seasonal variation of acute malnutrition in Northern Nigeria. It is unclear why there is a difference in MUAC values between ethnic Hausa and Fulani living in the same relatively small district; this may be inherent with a genetic basis, or due to differences in the diet and lifestyle of the two ethnic groups. The World Health Organisation growth reference study did not include children from pastoralist communities, those living in the Sahel, other desert areas, non-Bantu Africans or non-elite groups.

There are several limitations. First, we have only 1.5 years of data, so seasonality analysis has to be treated with extreme caution particularly with respect to the timing and magnitude of the effect. Second, although several additional variables which were collected concurrently such as market prices and the program coverage were not included in the final model because none were significant during the univariate analysis, there is the potential for variables that were not collected to co-vary between the outcome and predictor variables.

Third, the ecological validity and generalization of the findings to other populations and contexts needs to be demonstrated.

### Conclusions

This study shows that sentinel site surveillance is prone to observational bias and we present here estimates of the magnitude of this bias in North Nigeria. Although frequently repeated cross-sectional surveys with clusters randomised for each survey and a sample size representative of the population are likely to be more accurate, the effort and expense are probably unjustified in most situations. They may be feasible when MUAC alone is taken as the indicator of nutritional status. However, data collected during community screening to identify children in need to treatment for malnutrition using MUAC could also be used as a surveillance system to map the prevalence of GAM and SAM and observe its evolution in both time and space using a Geographic Information System based approach. The spatial data presented show that the area of this study, although relatively small, was not homogeneous with respect to nutritional status. Survey data gives a single prevalence figure for the whole area surveyed and variations within the area or “pockets” of malnutrition are not identified or targeted for relief; use of screening data for surveillance would have the advantage of addressing this problem at little additional cost.

Standardization tests [13, 21] should be performed systematically after training on anthropometric measurements, including MUAC, to confirm the ability of the staff to perform sufficiently precise and accurate measurements. Increased standardization and harmonization of the methods are required. This highlights the need for the development and implementation of a set of nutrition surveillance guidelines, with the intention of reducing the number of ad hoc surveys necessary, coordinating the surveys that are completed, and controlling the quality of the data that are collected and the reports produced. These higher-quality data will be more comparable between countries and more credible for use in implementing interventions and garnering support.
